# Chemical composition, antibacterial and antioxidant profile of essential oil from *Murraya koenigii* (L.) leaves

**Published:** 2014

**Authors:** Mini Priya Rajendran, Blessed Beautlin Pallaiyan, Nija Selvaraj

**Affiliations:** 1 *Department of Biotechnology, EDU-TECH Research Centre, EDU-TECH Educational and Research Institute, Melpuram, Pacode Post, Kanyakumari District, Tamilnadu, India – 629168.*; 2 *Department of Biotechnology, Udaya College of Arts and Science, Udaya Nagar, Vellamodi, Kanyakumari District, Tamilnadu, India.*

**Keywords:** *Antioxidant activity*, *Chemical composition*, *Essential oil*, *Murraya koenigii (L) leaves*

## Abstract

**Objective:** This study is designed to extract and examine chemical composition, antimicrobial and antioxidant activity of the hydro-distillated essential oil of *Murraya koenigii* leaves from the south region of Tamilnadu, India.

**Matherials and Methods:** Gas Chromatography (GC) and Gas Chromatography-Mass Spectrometry (GC-MS) analysis of the essential oil result was indicates the 33 different compounds representing 97.56 % of the total oil.

**Results:** Major compounds detected in the oil were Linalool (32.83%), Elemol (7.44%), Geranyl acetate (6.18%), Myrcene (6.12%), Allo-Ocimene (5.02), α-Terpinene (4.9%), and (E)-β-Ocimene (3.68%) and Neryl acetate (3.45%). From the identified compounds, they were classified into four groups that are oxygenated monoterpenes (72.15%), monoterpene hydrocarbons (11.81%), oxygenated sesquiterpenes (10.48%) and sesquiterpenes hydrocarbons (03.12%). The antibacterial activity of essential oil has pronounced by Disc Diffusion Method against various pathogenic microbes.

**Conclusion:** The oil has a maximum zone of inhibition ability against *Corynebacterium tuberculosis, Pseudomonas aeruginosa, Streptococcus pyogenes, Klebsiella pneumonia* and *Enterobacter aerogenes.* The antioxidant profile of the sample was determined by different test systems. In all the systems, essential oil showed a strongest activity profile within the concentration range.

## Introduction

Aromatherapy is a form of alternative medicine that uses volatile plant materials. It was known as essential oils and other aromatic compounds for the purpose of altering a person’s mind, mood, cognitive function or health. Evidence for the efficacy of aromatherapy in treating medical conditions remains poor, with a particular lack of studies employing rigorous methodology, lack of clinical evidence demonstrating efficacy against bacterial, fungal, or viral infections (Watt and Junca, 2008 ▶; Carson et al., 2006 ▶) but some research works revealed that the essential oils may have therapeutic potential (Ebert et al., 2007 ▶). 

Essential oils are volatile, natural and complex compounds characterized by a strong odor, formed by aromatic plants as secondary metabolites from many parts of plants, including the flowers, buds, seeds, leaves, twigs, bark, wood, fruit and roots (Burts, 2004 ▶; Dorman et al., 2007 ▶; Bakkali et al., 2008 ▶). In nature, it plays an important role in the protection of the plants as antibacterial, antiviral, antifungal and insecticides. Essential oils are valuable natural products used as raw materials in many fields, including perfumes, cosmetics, aromatherapy, medicine, Phytotherapy, spices and nutrition plus insecticides (Buchbauer, 2000 ▶). Many oils show antibacterial, fungicidal, relaxing, stimulating, antidepressant effect and may be a very effective therapeutic agent. Essential oils are known for their therapeutic properties hence, used in the treatment of various infections caused by both by pathogenic and non-pathogenic diseases (Hamid et al., 2007 ▶). Essential oils have been traditionally used for 35 differtent respiratory tract infections, and are used now a day as ethical medicines for colds. In the medical field, inhalation therapy of essential oils has been used to treat acute and chronic bronchitis and acute sinusitis (Boyd and Sheppard, 1970 ▶). Inhalation of vapors of essential oils or their individual volatile terpenes has a significant role in controlling the central nervous system (Boyd and Sheppard, 1970 ▶) and had an anti-inflammatory effect on the trachea and to reduce asthmatic symptoms (Burrows et al., 1983 ▶; Shubina et al., 1990 ▶; Frohlich, 1968 ▶). Essential oils and their individual aroma components also showed cancer suppressive inactivity when tested on a number of human cancer cell lines including glioma, tumors, breast cancer, leukemia and others (Hamid et al., 2007 ▶).

In the recent decades, there has been increasing interest in the use of plant antioxidants for scientific research as well as industrials purpose. This is mainly due to their strong biological activity, exceeding those of many synthetic antioxidants which have possible activity as promoters of carcinogenesis (Suhaj, 2006 ▶). Therefore, the need exists for safe, economical, powerful and natural antioxidants to replace synthetic ones (Tadhani et al. 2007 ▶). Antioxidants obtained from natural plant sources are more potent and safe due to their harmless nature. Medicinal plant and wild herbs are always under investigation due to these facts. Antioxidant activities of plants and plant aroma extracts obtained from spices have been investigated in various model systems (Nakatami, 1997 ▶; Mejia et al. 2002). Shahidi et al. (1992) ▶ have shown that the antioxidant effect of aromatic plants is due to the presence of hydroxyl groups in their phenolic compounds. The antioxidant ability of different plant extracts and oils can be measured using numerous assays. The various tested are based on specific features of the antioxidant activity (Chu et al., 2000 ▶).

*Murraya koenigii* (L.) Spreng or its common name curry leaf tree is a small strong smelling perennial shrub commonly found in forests as undergrowth. It was originally cultivated in India for its aromatic leaves and for ornament is normally used for natural flavoring in curries and sauces. Originated in Tarai regions of Uttar Pradesh, India. It is now widely found in all parts of India and it adorns every house yard of southern India and also it is now cultivated and distributes throughout the world. The plant is used in Indian system of medicine to treat various ailments (Kumar et al., 1999 ▶). Parts of the plant have been used as raw material for the traditional medicine formulation in India. This plant is known to be the richest source of carbazole alkaloids. It has been reported by authors that carbazole alkaloids present in *M.koenigii *(L.) Spreng and display various biological activities such as anti-tumor, anti-oxidative, anti-mutagenic and anti-inflammatory activities (Chakravarthy et al., 1982 ▶; Muthumani et al., 2010 ▶). *M. koenigii *leaves and roots can be used to cure piles and allay heat of the body, thirst, inflammation and itching. 

The aromatic leaves, which retains their flavor and other qualities even after drying, are slightly bitter, acrid, cooling, weakly acidic in tastes and are considered as a tonic, anthelmintic, analgesic, digestive, appetizing and are widely used in Indian cookery for flavoring food stuffs (Adebajo et al., 2004; Mandal et al., 2010 ▶). The phytoconstituents isolated so far from the leaves are alkaloids viz., mahanine, koenine, koenigine, koenidine, girinimbiol, girinimibine, koenimbine, O-methyl murrayamine A, O-methyl mahanine, isomahanine, bismahanine, bispyrayafoline and other phytoconstituents such as coumarin glycoside viz., scopotin, murrayanine, calcium, phosphorus, iron, thiamine, riboflavin, niacin, vitamin C, carotene and oxalic acid (Narasimhan et al., 1970 ▶; Narasimhan et al., 1975 ▶; Adebajo et al., 2006; Tachibana et al., 2003 ▶ Adebajo and Reisch, 2000 ▶). It also reported for anti-microbial, antioxidant (Singh and Sharma, 1978 ▶; Goutam and Purohit, 1974; Deshmukh et al., 1986 ▶). Essential oil composition of the leaves has been studied by various workers (Rana et al., 2004 ▶; Rao et al., 2011 ▶). The essential oil from leaves yielded di-α-phellandrene, D-sabinene, D-α-pinene, dipentene, D-α-terpinol and caryophyllene (Gopalan et al., 1984 ▶). This essential oil from *M. koenigii* leaves is reported to possess antioxiant, antibacterial, antifungal, larvicidal, anticarcinogenic, hypoglycemic, anti-lipid peroxidative, hypolipidemic and antihypertensive activity (Iyer and Uma, 2008 ▶; Goutam and Purohit, 1974).

The increasing importance of essential oils as pharmaceutical and aromatherapy assist besides their traditional role in cosmetics, not only as a potent ingredient but also as a fragrance donor. Moreover, it has opened up world wide opportunities for global marketing. As far as our literature survey could ascertain, the selected plant essential oils from *M. koenigii* serves as an important part in soap making ingredients, lotions, massage oils, diffusers, potpourri, scent, air fresheners, body fragrance, perfume oils, aromatherapy products, bath oils, towel scenting, spa's, incense, facial steams, hair treatments, and more. The purpose of this study was to evaluate essential oil of *Murraya koenigii* leaves as a new potential source of natural antioxidants and phenolic compounds. 

## Materials and Methods

**Isolation of the essential oil**

The fresh leaves of *M. koenigii* (100g) were collected from Kanyakumari District of Tamilnadu and submitted for 6 hours to hydro-distillation in 1000 ml distilled water using a Clevenger type glass apparatus (Purchased from ILE & Co Pvt Ltd, Madurai, India) for 6 hours. The obtained essential oil (1%) was dehydrating over anhydrous sodium sulfate and stored in dark sealed vials kept at 4^o^C for further analysis.

**Chemical composition analysis of essential oil**

*Gas Chromatography Analysis*

GC/FID (Gas chromatography with a

flame ionization detector ) analyses were carried out using a GC-14A with split/splitless-injector, FID and C-R6A-Chromatopac integrator (Shimadzu, Japan), a GC-3700 with FID (Varian, Germany) and C-R1B-Chromatopac integrator (Shimadzu). The carrier gas was hydrogen; injector temperature 250°C; detector temperature 320°C. The temperature programme was: 40°C/5 min to 280 °C/5 min, with a heating rate of 6°C/min. The columns were 30 m x 0.32 mm bonded FSOT-RSL-200 fuse silica, with a film thickness of 0.25 μm (Biorad, Germany) and 30 m x 0.32 mm bonded Stabi wax, with a film thickness of 0.50 μm (Restek, USA). Quantification was achieved using peak area calculations, and compound identification was carried out partly using correlations between retention times (Adams, 2001; Jennings, Shibamoto, 1980 ▶; Joulain and König, 1998 ▶; Kondjoyan and Berdaqué, 1996 ▶).

*Gas Chromatography and Mass Spectrometry (GC-MS) analysis*

For GC/MS measurements a GC-17A with QP5000 (Shimadzu), split/split less injector and Compaq ProLinea data system (class5k-software), a GCHP5890 with HP5970-MSD (Hewlett-Packard, USA) and ChemStation software on a Pentium PC (Bohm, Austria), a GCQ (Finnigan-Spectronex, Germany USA) and Gateway-2000-PS75 data system were used. The carrier gas was helium; injector temperature 250°C; interface-heating at 300°C, ion-source-heating at 200°C, EI-mode was 70eV electron energy. Mass spectra correlations were done using Wiley, NBS, NIST and our own library as well as published data (Adams, 2001; Joulain and König, 1998 ▶; Kondjoyan and Berdaqué, 1996 ▶).

**Antibacterial activity**

In the recent years due to an upsurge inantibiotic-resistant infections, the search for novel bioactive compounds to fight infections is an absolute necessity and in this regard, plant essential oils may offer a great potential and hope. Several studies have reported the efficacy of antibacterial obtained from the essential oils of various plant species (Mouhssen, 2001 ▶; Lee et al., 2007; Derwich et al., 2010 ▶). 

In this study, the essential oil was tested against ten bacteria, including Gram positive and gram-negative. *Corynebacterium pseudotuberculosis* (*ATCC-*43924),* Streptococcus pyogenes *(ATCC-12347), *Klebsiella pneumonia *(*ATCC-BAA-1706*)*, Pseudomonas aeruginosa* (ATCC-25348), *Enterobacter aerogenes *(*ATCC*-35029), *Vibrio cholera *(*ATCC*-14373), *Escherichia coli* (*ATCC*- 25922), *Salmonella enterica *(*ATCC*-29226), *Proteus mirabilis *(21721), and *Staphylococcus aureus* (*ATCC*-55804) obtained from the Department of Microbiology, EDU-TECH Research Centre. 

The disc diffusion method was employed for screening the antibacterial properties of isolated essential oil (Bauer, 1966 ▶). 

The bacteria strains were first grown on Muller Hinton medium at 37°C for 24 h prior to seeding on to the nutrient agar. A sterile 5-mm diameter filter paper disk (Whatman paper no.3) was placed on the infusion agar seeded with five hundred microliters of bacterial suspension, and oil extract was dropped on to each paper disk (40 μL per disk). A negative control was also included in the test using a filter paper disk (5mm size) saturated with DMSO (*dimethyl sulfoxide*) to check possible activity of this solvent against the bacteria assayed. A standard discs containing antibiotics (6mm size) were used as a reference control (Wayne, 2008 ▶). The treated plates were kept at 4°C for 30 min at room temperature to allow the diffusion of oil and then, incubated at 37°C for 24 h. The antibacterial activity was assessed by measuring the zone of growth inhibition (size in mm) surrounding the disks. Each experiment was carried out in triplicate.

**Antioxidant profile of essential oil**

*Scavenging effect on 2,2-diphenyl-1-picrylhydrazyl radical (DPPH)*

The radical scavenging capacity was determined according to the method described by (Mensor et al., 2001 ▶). 1.0 mL from 0.3 mM alcohol solution of DPPH was added to 2.5 mL from the samples with different concentration of *Murraya koenigii* leaf essential oil and of eugenol. The samples were kept at room temperature in the dark and after 30 min the optic density was measured at 518 nm. The optic density of the samples, the control and the empty samples were measured in comparison with ethanol. Ascorbic acid, rutin, butylhydroxy toluene (BHT) and butylated hydroxyl anisole (BHA) were used as positive control.

*Detection of hydroxyl radicals by deoxyribose assay*

The assay was performed as described elsewhere (Halliwell et al., 1987 ▶) with minor changes. All solutions were freshly prepared. 1.0 ml of the reaction mixture contained 28 mM 2-deoxy-D-ribose (dissolved in KH_2_ PO_4_/K_2_HPO_4 _buffer 10 mM, pH 7.4), 500 µL solution of various concentrations of the *Murraya koenigii* leaf essential oil or eugenol, 200 µM FeCl_3_ and 1.04 mM EDTA (1:1 v/v), 10 mM H_2_O_2_ and 1.0 mM ascorbic acid. After an incubation period of 1 h at 37 °C the extent of deoxyribose degradation was measured by the thiobarbituric acid (TBA) reaction. 1.0 mL of TBA (1 % in 50 mM NaOH) and 1.0 mL of trichloroacetic acid (TCA) were added to the reaction mixture and the tubes were heated at 100°C for 20 min. After cooling, the absorbance was measured at 532 nm against a blank (containing only buffer and deoxyribose). The percentage inhibition was calculated by the formula: 

I(%)=100 – (Abs_sample_/Abs_control_)x100

The IC_50 _(inhibitory concentration 50) value represented the concentration of the compounds that caused 50 % inhibition of radical formation (Halliwell et al., 1987 ▶). Quercetin was used as a positive control.

*Assay of Superoxide Radical Anion*

Superoxide anions were generated in an enzymatic system (xanthine-xanthine oxidase) and assayed by the reduction of nitroblue tetrazolium. The former comprised a solution of 100 μM xanthine, 60 μM nitroblue tetrazolium in 0.1M phosphate buffer at pH 7.4 and 0.07 U mL-1 xanthine oxidase in a total volume of 1 mL. This mixture was incubated at 25°C for 10 min and the optical density was recorded at 560 nm against a blank, which did not contain the enzyme (Robak and Gryglewski, 1988 ▶). In order to check the inhibitory effect of pimento oil on xanthine oxidase activity, the enzyme was assayed by measuring the formation of uric acid from xanthine (Robak and Gryglewski, 1988 ▶; Yuting et al., 2001 ▶). Concentrations of 50 μg/mL of *Murraya koenigii* leaf essential oil, eugenol, BHT were added to the samples before the enzyme was added. The percentage inhibition of xanthine oxidase was calculated by the formula: I(%)=100– (Abs_sample_/Abs_control_)x100

Evaluation of antioxidant activity in linoleic acid model system Linoleic acid emulsions was prepared by mixing 0.285 g of linoleic acid, 0.289 g of Tween 20 as emulsifier and 50 mL phosphate buffer (pH 7.2). The mixture was homogenized for 5 min according to Yen et al. (2003) ▶. The antioxidant was added at the final concentrations of 0, 0.002 and 0.005 % wt/vol of *Murraya koenigii* leaf essential oil, BHT 0.01 % was used as a control. The mixture was incubated in an oven at 37 °C for 10 d. The course of oxidation was monitored by measuring the conjugated Dienes formation (CD) and thiobarbituric acid reactive substances (TBARS). The antioxidant activity at the end of the assay time was expressed for each indicator as reduction percent of peroxidation (RP %) with the control containing no antioxidant (= 0 %). RP % = [(peroxidation indicator value w/o antioxidant) – (peroxidation indicator value with antioxidant) / peroxidation indicator value w/o antioxidant)] x 100. A higher percentage indicates a higher antioxidative activity. 

*Determination of conjugated diene (CD) formation*

Aliquots of 0.02 ml were taken at different intervals during incubation. After incubation, 2 mL of methanol in de-ionised water (60 %) were added, and the absorbance of the mixture was measured at 233 nm. The conjugated diene concentration was expressed in ml/mg in each sample. The results were calculated as 

CD = B x Vol/wt

Where, B - is the absorbance reading, Vol - denotes the volume (mL) of the sample and wt - is the mass (mg) of emulsion measured (Zainol et al., 2003 ▶).

*Determination of thiobarbituric acid reactive substances*

A modified thiobarbituric acid reactive substances (TBARS) method was used to measure the antioxidant activity of the essential oil in terms of inhibition of lipid peroxidation. 0.1 ml of sample was taken every day, from the emulsion, the following were sequentially added: the TBA-TCA solution (20 mm TBA in 15 % TCA). The mixture was heated in a 100°C water bath for 15 min and cooled at room temperature. After 2 ml of chloroform were added, the mixture was mixed and centrifuged at 2000 rpm for 15 min. The chloroform layer was separated and the absorbance of the supernatant was measured at 532 nm against a blank containing TBA-TCA solution. Malonic aldehyde standard curves were prepared by 1,1,3,3-tetramethoxypropane and TBARS were expressed as mg of malonic aldehyde/kg dry matter (Romero et al., 2004 ▶) 

**Statistical analysis**

The experimental data (in triple repetition) were included in an approximation model through polynomic dependences from fourth order. For all cases the plural correlation coefficient R^2^ was determined. The level of the concentration, which corresponds to 50 % of inhibition, was calculated according to this approximated dependence for which R^2^ was maximum level. The statistical processing of the data was carried out with the special software MATLAB (8.1).

## Results

The essential oil of Indian curry (*Murraya koenigii)* leaf collected from Kanyakumari district South Tamilnadu, India has been investigated for its composition and is responsible for the aroma and flavor associated with herbs, spices, and perfumes and strong antibacterial and antifungal activity when tested with microorganisms. The oils from the curry leaves were found to contain mostly oxygenated monoterpenes. Using GC and GC-MS 33 constituents were found with linalool (32.83%), elemol (7.44%), geranyl acetate (6.18%), myrcene (6.12%), allo-ocimene (5.02), α-terpinene (4.9%), and (E)-β-ocimene (3.68%) as the main compounds. From these identified compounds are classified in to 4 groups that are monoterpene hydrocarbons (11.81%), oxygenated monoterpenes (72.15%), sesquiterpenes hydrocarbons (03.12%) and oxygenated sesquiterpenes (10.48%) respectively (Table 1).

**Table 1:** Chemical composition (%) of the essential oil of *Murraya koenigii* leaves from south region of Tamilnadu, India

**Compound**	**Oil (%)**	**KI**
**β-Pinene**	0.3	974
**Myrcene**	6.12	991
**p-Cymene**	0.23	1023
**Limonene**	0.61	1027
**1,8-Cineole**	1.56	1030
**(Z)-β-Ocimene**	0.36	1036
**(E)-β-Ocimene**	3.68	1047
**γ-Terpinene**	0.21	1057
**α-Terpinolene**	0.3	1087
**Linalool**	32.83	1104
**1-Octen-3 yl acetate**	0.24	1108
**3-Octanyl acetate**	0.26	1112
**Allo-Ocimene**	5.02	1129
**α-Terpineol**	4.9	1191
**Nerol**	1.32	1228
**Carvone**	0.16	1244
**Linalyl acetate**	16	1258
**Lavandulyl acetate**	0.23	1293
**Myrtenyl acetate**	0.1	1325
**Neryl acetate**	3.45	1359
**Geranyl acetate**	6.18	1383
**Β-Elemene**	0.26	1393
**Z-Jasmone**	0.3	1402
**α-Gurjunene**	0.1	1411
**β-Caryophyllene**	1.3	1422
**Germacrene D**	0.2	1479
**α-Amorphene**	0.7	1483
**δ-Cadinene**	0.26	1522
**Elemol**	7.44	1552
**Viridiflorol**	0.92	1595
**γ-Eudesmol**	0.77	1635
**Β- Eudesmol**	1.15	1654
**Α- Eudesmol**	0.2	1657

Monoterpene Hydrocarbons 11.81 %

Oxygenated Monoterpenes 72.15 %

Sesquiterpene hydrocarbons 03.12 %

Oxygenated Sesquiterpenes 10.48 %

* Identification of volatile components is based on mass spectra value in reference to NIST 08 and standard libraries. Composition of grouped volatile compounds (%)

The effectiveness of the *M. koenigii* leaf essential oil is demonstrated by the size of the inhibition zone around the filter paper disk on microbial growth, which is typically expressed as the diameter of the inhibition zone in millimeter. 

**Fig.1:**
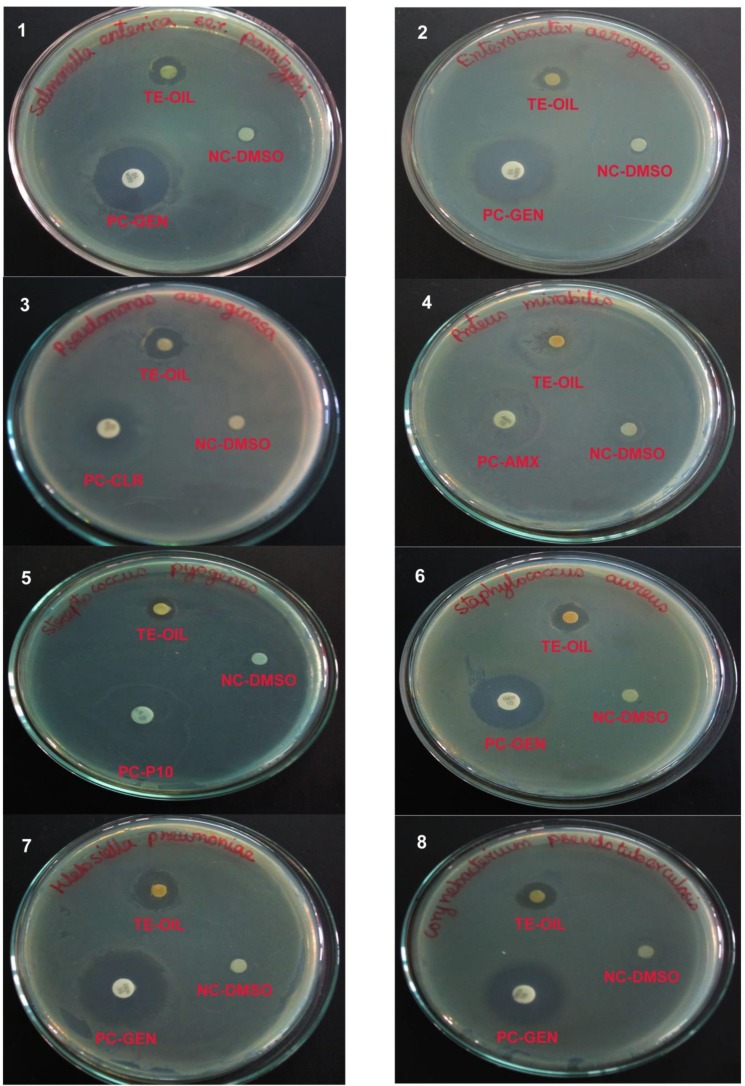
Antibacterial activity of *Murraya koenigii* leaf essential oil against 8 selected bacteria by disc diffusion method reference with antibiotics. TE-OIL: Test Oil (*Murraya koenigii* leaf essential oil, NC-DMSO: Negative Control – Dimethyl Sulfoxide, PC-GEN: Positive Control – Gentamicin, PC-CLR: Positive Control – Clarithromycin, PC-AMX: Positive Control Amoxicillin, PC-P10: Positive Control – Penicillin. Bacterial species tested for antibacterial effect of oil - 1. Salmonella enterica 2. Enterobacter aerogenes 3. Pseudomonas aeruginosa 4. Proteus mirabilis 5. Streptococcus pyogenes 6. Staphylococcus aureus 7. Klebsiella pneumoniae 8. Corynebacterium psedotuberculosis

The remarkable results obtained in the antibacterial study with the comparison of positive and controls are shown in Figure 1 and Table 2. The results indicated that the oil of *M. koenigii* with the strongest inhibition zone against *Proteus mirabilis* (18mm), *Staphylococcus aureus*, *Corynebacterium pseudotuberculosis *(15 mm),* Klebsiella pneumoniae *(15 mm) *Pseudomonas aeruginosa* (14mm), *Enterobacter aerogenes *(13 mm) and the moderate level zone of inhibition observed against in* Salmonella enterica *(11mm),* Streptococcus pyogens *(10 mm) respectively.

**Table 2:** Antibacterial Activity of essential oil compared with selected antibiotics tested on ten strains of bacterial pathogens

**Sl.No.**	**Tested Bacteria**	**Zone of inhibition (mm)**		
		**EO**	**NC**	**PC**
**1**	*Corynebacterium psedotuberculosis*	15	5	20 (GEN10)
**2**	*Streptococcus pyogenes*	10	6	24 (P10)
**3**	*Klebsiella pneumoniae*	15	5	24 (GEN10)
**4**	*Pseudomonas aeruginosa*	14	5	12 (CLR15)
**5**	*Enterobacter aerogenes*	13	6	20(GEN10)
**6**	*Salmonella enterica*	11	6	22 (GEN10)
**7**	*Proteus mirabilis*	24	6	23 (AMX10)
**8**	*Staphylococcus aureus*	16	5	20 (GEN10)

Note: EO-Essential Oil of *Murraya koenigii* leaves; NC - DMSO as a Negative Control (5mm size of disc from whatman no.3 filter paper); PC-Positive Control : AMX10 - Amoxicillin (10mg/disc); CLR15 - Clarithromycin (15mg/disc); GEN10- Gentamicin (10mg/disc); P10 - Penicillin (10mg/disc)

Results of the investigations of the DPPH radical-scavenging activity are as follows: DPPH is a stable free radical and accepts an electron or hydrogen radical to become a stable diamagnetic molecule. Table 3 shows the concentrations of the essential oil, and of eugenol, rutin, ascorbic acid, BHT and BHA that resulted in a 50 % inhibition of the free radical DPPH (IC_50_). Following the decrease in antioxidant activities, the components under study were arranged in the order: BHA > eugenol > essential oil > ascorbic acid > BHT > rutin. IC_50_ values were approximated with statistical significance p≤0.01 and with high regression coefficients.

**Table 3.** Effect of the test compounds in the DPPH assay

**Test compound IC50** * ** R2** **	**Test compound IC50** * ** R2** **	**Test compound IC50** * ** R2** **
**Essential Oil**	1.79	0.998
**Eugenol **	1.26	0.999
**Rutin **	14.65	0.991
**Ascorbic acid **	4.20	0.994
**Butylated hydroxytoluene (BHT) **	4.47	0.998
**Butylated hydroxyanisole (BHA) **	1.12	0.996

* Concentration (μg/mL) for a 50 % inhibition

** R2 – correlation coefficient

**Table 4:** Effect of *Murraya koenigii* leaf essential oil, eugenol and BHT on xanthine oxidase activity

**Compound tested**	**A295 nm**	**% inhibition**
**Control **	0.310	-
**Essential oil (** **50 μg/mL)**	0.078	74.83
**Eugenol (50 μg/mL)**	0.120	61.29
**BHT (50 μg/mL)**	0.096	69.03

Note: BHT- it describes Butylated Hydroxytoluene

**Table 5:** Superoxide anion scavenging activity of *Murraya koenigii* leaf essential oil, eugenol and BHT

**Compound tested**	**A560 nm**	**% inhibition**
**Control **	0.295	-
**Essential oil (** **(50 μg/mL)**	0.012	95.93
**Eugenol (50 μg/mL)**	0.051	82.71
**BHT (50 μg/mL)**	0.082	72.20

Note: BHT- it describes Butylated Hydroxytoluene

## Discussion

The essential oils from aromatic plants are for the most part volatile and thus, lend themselves to several methods of extraction such as hydrodistillation, water and steam distillation, direct steam distillation, and solvent extraction of which hydro-distillation, steam and steam/water distillation are the most common method of extraction (Bowles, 2003 ▶; Margaris et al., 1982 ▶; Surburg and Panten, 2006 ▶). Typically, essential oils are clear, however; there are few exceptions. For example, the essential oil of *M. koenigii *leaves having dark yellow color.

An essential oil is generally steam or hydro-distilled from flowers, leaves, bark and roots of plants and trees and are the compounds responsible for the aroma and flavor associated with herbs, spices, and perfumes. The formation and accumulation of essential oils in plants have been reviewed by Croteau (1986) ▶, Guenther (1972) ▶ and Runeckles and Mabry (1973) ▶. Chemically, the essential oils are primarily composed of mono and sesquiterpenes and aromatic polypropanoids synthesized via the mevalonic acid pathway for terpenes and the shikimic acid pathway for aromatic polypropanoids. Essential oils of plants in the family Rutaceae are often composed of mono and sesquiterpenes. In the present investigation, the oil was dominated by the linalool (32.83%) and monoterpenic ester linalyl acetate (16%) but the previous report of same plant species growing in Bangladesh dominated by the 3-carene (54.22%) and linalool (0.19%) was present as minor compound (Chowdhury et al., 2008). Other major components are Elemol (7.44%), Geranyl acetate (6.18%), Myrcene (6.12%), Allo-Ocimene (5.02%), α-Terpineol (4.9%), (E)-β-Ocimene (3.68%), Neryl acetate (3.45%) respectively.

Linalool is reported to possess antibacterial, insect-repelling and toxic activities (Cardile et al., 2009 ▶). Linalool due to its floral pleasant scent has been many commercial applications in perfumed hygiene products and cleaning agents including soaps, detergents, shampoos, and lotions, etc. It is also used as a chemical intermediate in synthesis of vitamin E. Linalool is used as a flea and cockroach insecticide (Klimánková et al., 2008 ▶; Ahmed et al., 2000 ▶). One of the research reports was found that inhaling linalool can reduce stress in lab rats so the findings could form the basis of new blood tests for identifying fragrances that can soothe stress (Nakamura et al., 2009 ▶).

Hydroxyl radical-scavenging activity investigations showed following data: Hydroxyl radicals were generated in a reaction mixture containing ascorbate, hydrogen peroxide and iron III-EDTA at pH 7.4 and measured by their ability to degrade the sugar deoxyribose (Halliwell et al., 1987 ▶; Aruoma et al., 1989 ▶). The presence of the essential pimento leaf oil in the reaction mixture protected deoxyribose against degradation by eliminating the highly reactive hydroxyl radicals (OH•). Results from this study characterized *Murraya koenigii* leaf essential oil as a strong OH•-scavenger in rivaltry with 2-deoxy-D-ribose, the effect intensifying with the increase of concentration and reaching as high as 87.27 %, while *Murraya koenigii* leaf essential oil major component linalool was performed 91.20 % inhibition of OH• at 0.6 μg/mL concentration. The antioxidant activity of quercetin have performed 77.8 % at 20 μg/mL was substantially weaker. The three studied antioxidants were arranged by their antioxidant effect (expressed as IC_50_), in descending order, as follows: eugenol - 0.06 µg/mL (R^2^ = 0.989), essential oil - 0.29 µg/mL (R^2^ = 0.999), quercetin - 4.61 µg/mL (R^2^= 0.834). When Fe^3+^ -ions are added to the reaction mixture as FeCl_3_ instead of EDTA complex, some of the iron ions form complexes with deoxyribose. The Fe^3+^ may be subsequently reduced by ascorbate to Fe^2+^, which in turn may remain bound to deoxyribose and further react with H_2_O_2_. The reaction generates the necessary OH•, which immediately triggers the degradation of deoxyribose in a site-specific manner. Only molecules that are able to chelate ions and make them inactive may inhibit the degradation of deoxyribose.

The essential oil, eugenol and quercetin as scavengers of OH• and manifests chelative properties, most expressive in the case of eugenol. Like most radicals, OH• can be neutralized by a hydrogen atom. 

 The evaluation of pimento oil’s scavenging activity with respect to the two radicals in concern – DPPH and OH•, showed that it was a more effective scavenger of OH•-radicals – the respective IC_50_ values were 0.29 μg/ml for OH• against 1.79 μg/ml for DPPH.

The superoxide anion scavenging activity results can be discussed as follows: Superoxide is biologically important since it can be decomposed to form stronger oxidative species such as singlet oxygen and hydroxyl radicals (Korycka-Dahl and Richardson, 1978 ▶). Superoxide anions indirectly initiate lipid oxidation serving as precursors of singlet oxygen and hydroxyl radicals (Robak and Gryglewski, 1988 ▶). Xanthine-xanthine oxidase is the system, which is often used as a generator of superoxide radicals. The results from this study showed that the essential *M.koenigii *leaf oil, eugenol and BHT have inhibitory effect on antioxidant enzyme activity (Table 4). 

At 50 μg/mL concentration, the xanthine oxidase inhibition power of the studied components arranged them in the following order: essential oil > BHT > eugenol. By inhibiting xanthine oxidase activity of *Murraya koenigii* leaf essential oil cut down the generation of superoxide radicals. The superoxide anion scavenging activity of *Murraya koenigii* leaf essential oil, eugenol and BHT is presented in Table 5.

The decrease in the absorption at 560 nm suggested superoxide radicals’ scavenging, being most significant when the essential oil was added to the reaction mixture – 95.93 %. Superoxyde dismutase 100 U/mL with inhibiting effect 77.8 % was used as a standard. The results achieved in the study revealed two ways of influence of essential oil and eugenol on xanthine-xanthine oxidase function – they acted as xanthine oxidase inhibitors and superoxide scavengers, thus confirming the interpretation of (Jubert et al., 2004 ▶).The evaluation of antioxidant activity in a linoleic acid model system was as follows: For the task of evaluating the inhibitory effect of essential oil on lipid peroxidation, a model system of linoleic acid emulsion was applied. The antioxidant capacity was estimated both at the early stages of linoleic acid autoxidation and later, after the emergence of secondary oxidation products, expressed as malonic aldehyde content. Two indicators were referred to, corresponding to a different degree of lipid peroxidation – conjugated diene formation and TBARS. The effect of the essential *Murraya koenigii* leaf oil on lipid peroxidation was assayed at body temperature, 37°C.

For the determination of conjugated diene (CD) formation the following results were found: An increase of essential oil concentration from 0.002 to 0.005 % caused a corresponding increase of the inhibitory effect of the component on lipid peroxidation, for the entire study period Linoleic acid peroxidation leading to CD formation was most intensive on the fifth day of acid incubation. At this stage of incubation, with 0.005 % *Murraya koenigii* oil added, significant inhibition of the process was achieved - 53.32 %, compared to the 35.21 % inhibition realized with BHT. The most effective inhibition of the process was observed on the eighth day of the study, when 0.005 % concentration of *Murraya koenigii* leaf essential oil lead to an inhibitory effect greater than that of 0.01 % BHT - 65.47 % and 61.90 %, for the two substances respectively.

Finally, the determination of thiobarbituric acid reactive substances can be discussed as follows: with the second reference used for estimating the presence of secondary derivatives from linoleic acid oxidation (TBARS), maximum accumulation of malonic aldehyde was found. It ws also found, as the case above, on the fifth day of linoleic acid incubation, suggesting that the process ran in a manner nearly analogous to the formation of conjugated dienes. The strongest antioxidant action of the compounds under study was exercised on the eighth day of the study, with the inhibitory effect of essential *Murraya koenigii* leaf oil being almost equal to that of the synthetic standard BHT; moreover, the effect was executed at a concentration twice as low than that of BHT (0.005 %). The inhibition of lipid peroxidation by *Murraya koenigii* oil obtained 72.98 % compared to 76.47 % inhibition realized by BHT.

Therefore, based on this investigation, it was concluded that the chemical composition and antioxidative properties of the essential leaf oil of *Murraya koenigii* from South Tamilnadu, India that linalool, elemol, geranyl acetate, myrcene, allo-Ocimene and α-terpinene are the main components with such capacity. The presence of major compounds in *M.* *koenigii *is not reported from elsewhere, completely differs from those reported by Raina et al. (2002) ▶, Walde et al. (2005) ▶ and Chowdhury et al. (2008). The undiluted essential oil exhibited strong antibacterial activity when tested with microorganisms. In comparison with the antibiotics as positive control they are expressing the remarkable activities on *Proteus mirabilis *compared in Amoxicillin (10mg/disc)*,* *Staphylococcus aureus* compared in Gentamicin (10mg/disc), *Corynebacterium pseudotuberculosis* compared in Gentamicin (10mg/disc),* Klebsiella pneumonia *compared in Gentamicin (10mg/disc)*,* *Pseudomonas aeruginosa* compared in Clarithromycin (15mg/disc), *Enterobacter aerogenes* compared in Gentamicin (10mg/disc) and the moderate level zone of inhibition observed against in* Salmonella enterica* compared in Gentamicin (10mg/disc),* Streptococcus pyogenes* compared in Penicillin (10mg/disc). Based on this finding, it can be concluded that the essential oil of *M. koenigii* have the bioactive potential and it will be the prominent antibiotic therapy for tested microbes. This oil may be studied for any bioactive properties for therapeutic uses and also may serve as a useful source of flavors and fragrances. *Murraya koenigii* essential oil manifested greater antiradical activity with respect to OH• in comparison with DPPH radicals, attested by the respective IC_50_ values. The inhibitory capacity of *Murraya koenigii* oil on OH• exceeded that of quercetin. The oil was a Fe^3+^chelator, too, thus preventing the initiation of hydroxyl radicals.

*Murraya koenigii* essential oil inhibited xanthine oxidase activity, which caused a decrease of the generation of superoxide radicals. Moreover, there was a second mechanism of action involved, the scavenging of superoxide radicals. *Murraya koenigii* essential oil was capable of an effective inhibition of both the conjugated diene formation and the generation of secondary products from lipid peroxidation, carried out at a concentration twice lower than that of the reference antioxidant BHT. The use of some methods to determine the antioxidative properties of medicinal plant essential oils confirms the findings that antioxidative capacity detected by only a single method should be interpreted with some caution (Mantle et al., 1998 ▶). The established proven antioxidant properties of *Murraya koenigii* leaf essential oil thus broaden the scope for its implementation in food industry and medicine.
